# Emerging Viral Pathogens in Sturgeon Aquaculture in Poland: Focus on Herpesviruses and Mimivirus Detection

**DOI:** 10.3390/v13081496

**Published:** 2021-07-29

**Authors:** Magdalena Stachnik, Marek Matras, Ewa Borzym, Joanna Maj-Paluch, Michał Reichert

**Affiliations:** Department of Fish Diseases, National Veterinary Research Institute, 24-100 Puławy, Poland; marek.matras@piwet.pulawy.pl (M.M.); ewa.borzym@piwet.pulawy.pl (E.B.); joanna.maj@piwet.pulawy.pl (J.M.-P.); reichert@piwet.pulawy.pl (M.R.)

**Keywords:** sturgeon, herpesvirus, AciHV-1, AciHV-2, sNCLDV, mimivirus, AciV-E

## Abstract

Recently, Poland has become a leading producer of sturgeon meat and caviar in Europe and is one of the largest in the world. The growing importance of this branch of aquaculture means that diseases of these fish, especially viral ones, are becoming the object of interest for ichthyopathologists. In recent years, there have been increasing reports of health problems in the dynamically developing sturgeon farming. The greatest risk appears to be emerging infectious diseases that are caused by viruses and that can become a serious threat to the development of the aquaculture industry and the success of sturgeon restitution programs undertaken in many European countries, including Poland. In this paper, an attempt was made to determine the spread of the two most important groups of viruses in Polish sturgeon farming: These include the herpesviruses and sturgeon nucleocytoplasmic large DNA viruses (sNCLDV), in particular, mimiviruses. In the years 2016–2020, 136 samples from nine farms were collected and tested by using the WSSK-1 cell line, PCR and Real Time PCR methods. All results were negative for herpesviruses. Out of the samples, 26% of the samples have been tested positive for mimiviruses. Sanger sequencing of mimiviruses demonstrated their affiliation with AciV-E. The sequence characterization confirmed the presence of both V1 and V2 lineages in Polish fish facilities, but variant V2 seems to be more widespread, as is observed in other European countries.

## 1. Introduction

Sturgeons (*Acipenseridae*) in recent decades have experienced a variety of negative effects of human activity which resulted in a significant decline in its population in the world. Today, almost all species of sturgeon are threatened with extinction and more than half are assigned as critically endangered species on IUCN 2000 Red List [1]. In the past 30 years, over-exploitation of wild sturgeon stocks caused the main sturgeon fisheries—in the Baltic Sea and in the most important production area, the Caspian Sea—to seriously decline. Strict international and domestic regulations are now in place to further prevent the decline of those critically endangered species and, in 2011, international trade from all the major stocks of sturgeon was suspended. As wild sturgeon stocks and caviar production reduced, global demand for caviar increased which sent prices soaring and this paved the path for sturgeon aquaculture to enter the arena. In order to repopulate the natural environment and to supply an increasing market for caviar as well, sturgeon farming has been developed in many countries. The largest world producers of meat and caviar were China, Italy, France and Russia. Recently, Poland has become the third largest caviar producer in the world and the second in Europe [2]. According to CITES reports, Poland recorded the most rapid growth, with caviar export volumes standing at virtually nothing until 2015 to exporting nearly 13 tonnes in 2018; the main farmed sturgeon species were the Siberian sturgeon and Russian sturgeon [2,3].

A few centuries ago, sturgeons were widespread in Europe and, today, the current state of the European population is dramatic. Among other things, the Baltic sturgeon (*Acipenser sturio*) that once occurred in Polish waters is considered an extinct species. Several European countries, such as Poland, France and Germany, have already started reintroduction programs. The Baltic sturgeon population found in the Baltic Sea was considered extinct in the mid-twentieth century. In Poland, work on the restitution of the Baltic sturgeon species is carried out since 2004. The material used for this purpose include fertilized eggs and hatch obtained from the most closely related and genetically similar population of the Atlantic sturgeon (*Acipenser oxyrinchus*) from the coast of Canada [4,5].

Fish farming always faces pathogenic agents which provoke disease and can devastate the cultured population. The most serious sturgeon diseases are caused by viruses from Herpesviridae and sturgeon Nucleocytoplasmatic Large DNA Viruses (sNCLDV), in particular, the Iridoviridae family which is recently classified unofficially as Mimiviridae. They exist in the wild populations and are introduced into farms when genitors are captured, often with dramatic effects for farmed fish. To date, more than 10 different specific viruses have been found in sturgeons [6]. Almost all of them were discovered in species native to North America, both in the USA and Canada, as well as in Europe where fish from America were introduced [7,8,9,10,11,12]. Lately, some cases of infection with herpesvirus, iridovirus or sNCLDV were found in non-American sturgeon species in Asia and Europe [13,14,15,16,17]. In recent years, there have been more and more disturbing reports of health problems of viral etiology in wild and farmed sturgeon [18,19,20,21,22,23,24]. Emerging infectious diseases caused by herpesviruses and mimiviruses seem to be of greatest importance [6,7]. This phenomenon, which is dangerous for the development of this branch of aquaculture, poses a serious threat to the success of fish species restitution programs undertaken in European countries.

This paper describes the results of the first screening of different viral agents in the collection of samples from sturgeons in Poland, with particular emphasis on mimivirus and herpesviruses. Various diagnostic methods, such as cell culture and PCR, were used to increase the effectiveness of viral infection monitoring. For the first time in Poland, the presence of emerging mimivirus in both healthy and infected fish was confirmed by PCR. Other pathogens, including herpesviruses, have not been isolated. Here, we report the first molecular data supporting the existence of two different variants of mimivirus in fish facilities in Poland. These epidemiological findings may help implement effective sturgeon health control strategies by identifying the risks that threaten this important and rapidly growing aquaculture sector in Poland.

## 2. Materials and Methods

**Figure 1:**
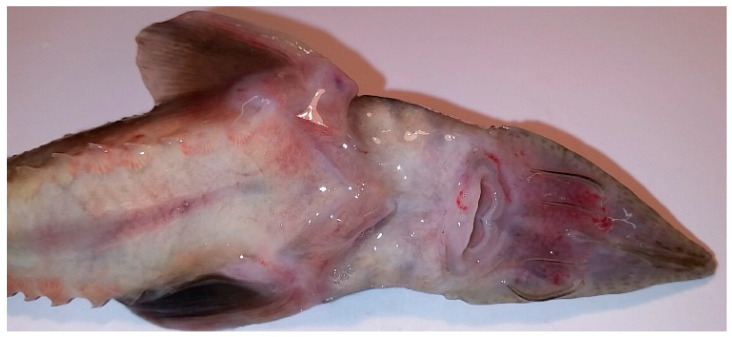
Siberian sturgeon with multiple haemorrhages (farm 5).

**Figure 2:**
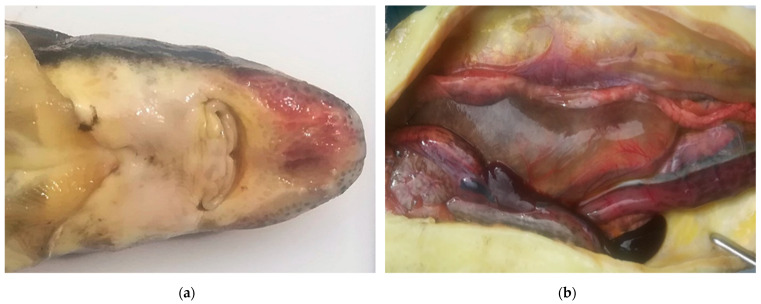
Russian sturgeon from farm 8 showing clinical signs: (**a**) haemorrhage on the rostrum (**b**) splenomegaly, intestinal haemorrhage and swim bladder filled with yellowish fluid.

An amount of 136 fish samples were collected from 9 facilities in 2016–2020 and they were part of the material for standard health tests performed as health surveillance on farms. All fish (7 batches of *Acipenser baerii* and 2 batches of *Acipenser gueldenstaedtii*) originated from Polish brood stock. With the exception of farm 5 and 8, all fish were clinically healthy and no higher mortality or severe disease symptoms were reported (Figure 1 and Figure 2a,b).

Pathological changes in fish from farm 5 have been observed during clinical study in the laboratory. Siberian sturgeon (PL1719-PL1729) had multiple petechial haemorrhages on the ventral part of the rostrum, around the mouth and on the abdominal and lateral body surfaces (Figure 1). Some fish had extremely pale livers. The bacteriological and parasitological tests showed negative results and, thus, it was decided to test the samples for the presence of viruses. Farm 8 was the only facility where apathy, lack of appetite, uncoordinated swimming and a mortality rate of 30–50% in some tanks with Russian sturgeon were observed. The batch of fish from farm 8 (PL1801-PL1806) had haemorrhages on the rostrum and on the abdominal and lateral body surfaces, enlarged spleen and fluid in the peritoneal cavity and swim bladder (Figure 2a,b). Bacteriological and parasitological tests revealed the presence of bacteria (*Aeromonas hydrophila*, *Shevanella putrefaciens* and *Flavobacterium* sp.) in the skin and gill tissues and larvae of eye fluke *Diplostomum spathaceum* in lens of the diseased fish. As parasites and bacteria were excluded as main causes of the disease and increased mortality, the affected tissues from fish were sampled for virological examination.

A series of all collected samples from every batch was analysed for the presence of viruses, although most of the fish did not exhibit clinical signs and bacteriological and parasitological tests were negative. Fresh tissues of various organs were either analysed by using cell culture or fixed in 90% ethanol and taken for subsequent DNA extraction.

### 2.1. Virus Isolation on WSSK-1 Cell Line

Internal organs (spleen, kidney and heart) from each sturgeon were pooled, homogenized, diluted 1:10 in Leibovitz media supplemented with of antibiotic/antifungal solution and 10% of foetal calf serum (Invitrogen) and clarified by centrifugation. Samples were inoculated on 24-well plates with 24 h old white sturgeon skin cell line WSSK-1 (kindly provided by Prof. Ronald P. Hedrick, UC, Davis, CA, USA). These inoculated cell cultures were incubated at 20 °C for 7–10 days, with daily examination of monolayer for a cytopathic effect (CPE) [10,25]. On each plate, a well with WSSK-1 cells without inoculum was used as a control to compare the differences between infected and uninfected cells. Observation to identify morphological changes of cells, such as pyknosis of nuclei and cellular fusion (syncytia), was performed according to the method [25] by using inverted phase contrast microscope (Olympus, Japan) at magnifications of 200×–400×. After first week incubation, samples with no CPE effect were re-inoculated into a new cell culture. If no CPE was observed at the end of the fourth week, the sample was considered as negative for virus isolation.

### 2.2. Nucleic Acid Extraction for PCR Detection and Sequencing

DNA was extracted from organs pooled from each fish (fins, gills, spleen and kidney) in 90% ethanol by using the Qiamp DNA Mini kit (Qiagen, Hilden, Germany). The list of oligonucleotides and probes used for testing is provided in the Table 1. As a control of the isolation quality and integrity of the extracted nucleic acids, real-time PCR was performed targeting the mitochondrial 16S rRNA gene of sturgeon. Primers Aci16SFw, Aci16SRv and the probe Aci16SPb were used at 200 nM each in a final reaction of 20 μL containing 12.5 μL of the Quantitect Probe PCR Kit (Qiagen) and 5 µL of DNA. An initial activation step of 15 min at 95 °C was performed, followed by 40 cycles of 15s at 94 °C and 60s at 60 °C [15]. All DNA samples were examined by real-time PCR to obtain amplification targeting the MCP gene region of mimivirus with primers oPVP346 and oPVP347 [13]. Reactions were performed in 25 µL with 400 nM each primer and 150 nM probe tqPVP20 for the Taqman assay (Quantitect Mastermix; Qiagen) according to a following protocol: 15 min at 95 °C, 40 cycles of 15 s at 94 °C and 60 s at 60 °C. A positive control was obtained from the sample PL1601, first positive and amplified with primers oPVP339 and oPVP342. The DNA of samples determined as positive by the real time method was used for conventional PCR method with a set of primers A or D to amplify the 641 bp or 1344 bp for sequencing of MCP gene fragment (Table 1).

PCRs were performed in 50 µL with 500 ng total DNA, 0.4 µM each primer of a pair, 1.5 mM MgCl2, 200 µM dNTP and 1 U Platinium Taq polymerase (Invitrogen) under the following conditions: Initial denaturation step at 95 °C for 5 min, followed by 35 cycles with denaturation at 95 °C for 30 s; annealing at 54 °C for 30 s; extension at 72 °C for 30 s and final extension at 72 °C for 10 min. For detection of sturgeon herpesviruses, PCRs developed by Kurobe and co-authors were used with a set of primers, 308 and 309, to amplify the 322-bp fragment of the AciHV-1 termination gene and primers TermF2 and Termsal-3Rdeg, binding a 531-bp fragment of the AciHV-2 terminase gene [8]. The herpesvirus PCR conditions were the initial denaturation step of 95 °C for 5 min, then 40 cycles of 95 °C for 30 s, 40 °C for 30s and 72 °C for 1 min, followed by a final extension step at 72 °C in the 50 μL volume of the reaction mixture of Platinum Taq DNA polymerase (Invitrogen) reagents. Each stage of amplification was performed by Biorad thermocycler. The PCR products were separated on 2% agarose gels and observed by a transilluminator after staining with a 1% Simply Safe Reagent (EURX) for 30 min. The sequencing of PCR products (Sanger Dideoxy Method) was performed by a commercial company (Genomed, Warsaw, Poland).

### 2.3. Sequence Analysis and Phylogenetic Tree Analysis

The DNA sequences obtained from Sanger sequencing were assembled and the consensus sequences were edited using Genenious 10 software [26]. Then, they were aligned with the ClustalW function of MEGA X software [27]. Phylogenetic trees showing the relationships of the sturgeon mimiviruses were generated based on the partial MCP gene using the neighbor-joining algorithm. The similarity of the nucleotide sequences of the detected mimivirus MCP gene fragments and maximum-likelihood analyses were performed by using the Tamura Nei substitution model implemented in the MEGA X after trimming sequences to the shortest one [28]. The reliability and robustness of the phylograms were tested by bootstrap analysis with 1000 replications. The values greater than 80% were considered as strong evidence for robust phylogenetic groupings.

## 3. Results

### Virus Isolation on WSSK-1 Cell Line and PCR Detection and Sequencing

Incubation of all samples on the plates with the WSSK-1 cell line was unsuccessful. No cytopathic effect on the first passage and for the following passages of cells was detected. Nucleic acid extraction for molecular virus detection was correct and provided good quality material for further analysis. A positive signal of the presence of the 16S host gene was obtained for all tested samples with a Ct value in the range 15–23 (data not shown). The presence of herpesviruses AciHV-1 and AciHV-2 was not confirmed in any of the samples. In contrast, among the 136 analysed DNA samples (from pooled organs from each fish), 36 samples were positive by Real Time PCR for the presence of mimivirus and Ct values ranged from 18–36 (data not shown). The description of the samples and the results of mimivirus tests are presented in Table 2.

**Figure 3:**
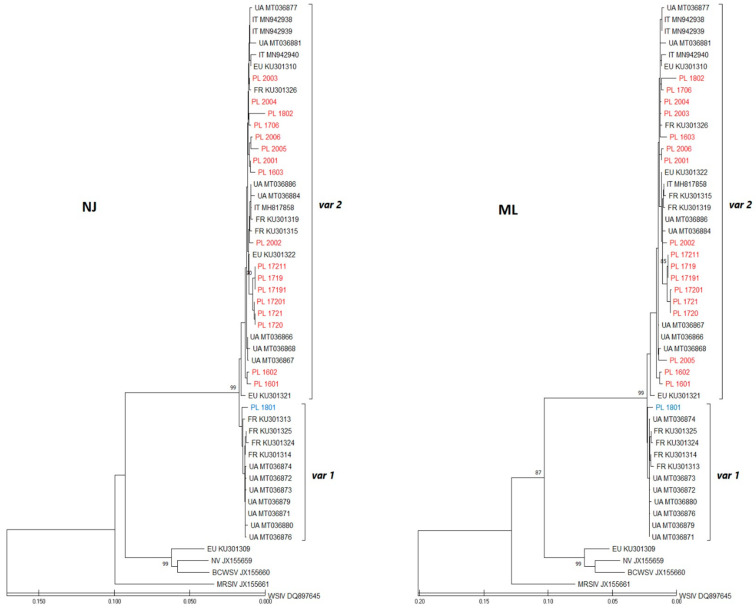
Analysis of the partial mimivirus major capsid protein (MCP) sequences using the neighbor-joining (NJ) and maximum-likelihood methods (ML). For each sequence, the country of origin is indicated (two-letter country code: PL—Poland; UA—Ukraine; IT—Italy; FR—France; EU—other European countries). Polish isolates are marked in blue (*var1*) and red (*var2*). Bars indicate the scale of genetic distance between sequences.

Conventional PCR on set primer A was performed for result confirmation (636 bp) and on set primer D for sequencing (1344 bp). Unfortunately, of the 36 positive samples in Real Time PCR, only 18 were successfully amplified on primer set D and deposited in GenBank with the accession numbers in Table 2. The mimivirus prevalence on individual farms ranged from 0 to 73%. This depended mainly on the fact of whether the samples came from standard surveillance tests or were delivered due to the occurrence of health problems in a given facility (farm 5 in 2017 and farm 8 in 2018). By comparing the years of research, it can be observed that the annual average level of infection per year varied from 15 to 33% and was lower the more samples were tested. Most of the infected fish belonged to the species *A. baerii* (34 out of 136 samples) and only two Russian sturgeon were positive for mimivirus presence (Table 2). After trimming to 1134 nt, the analysis of the partial protein of the main mimivirus capsid of Polish sequences was performed. The sequences obtained in this study were compared to sturgeon mimivirus sequences from other countries and identities ranging from 97% to 100% were found. All shared only 87% of their identity with the European variant of NV-sequence EU KU301309, which was clustered with American sturgeon nucleocytoplasmic large DNA viruses-sNCLDV (Figure 3).

**Figure 4:**
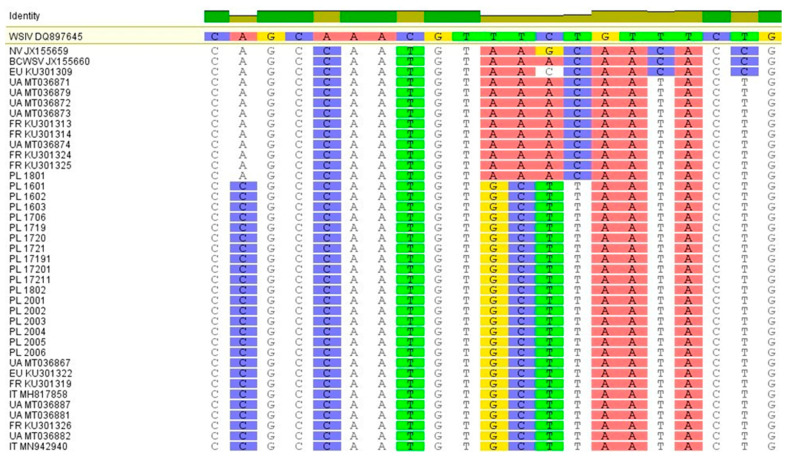
Aligned Polish sequences with highlighted motifs characteristic for American strains (*var1*-AAACAATA) and European variant (*var2*-GCTTAATA) of mimivirus.

During both analyses and on the ML and NJ phylogenetic trees, Polish sequences, together with other Europeans available in the GenBank database, were grouped into two genogroups that are correlated with the allele type of variant 1 or variant 2 in the MCP protein as described by Pallandre and co-authors [20,21] as *var1* or *var2* (Figure 3). The first genogroup included sequences with a *var1* motif from France and Ukraine and only one sequence from Russian sturgeon from Poland (PL1801). Within this group, the Polish sequence was quite divergent from Ukrainian and French. In the second genogroup, all the sequences had a *var2* motif and the Polish sequences were almost identical to sequences from other countries. In this group, the isolates PL1719-PL17211 had the least divergence and came from sick fish from farm 5 associated with the outbreak of mimivirus and increased mortality. In general, mimivirus infection was confirmed in six out of nine farms, with *var2* occurring more frequently in fish than *var1*. Only one sequence from *A*. *gueldenstaedtii* (PL1801) had one single nucleotide polymorphism (SNP) difference in terms of sequence characteristic for strains closely related with American isolates (*var1* AAACAATA) [20,21]. All the remaining sequences were assigned to the European variant (*var2* GCTTAATA), including sample PL1802 from the same farm and species as PL1801 (Figure 4). The presence of different virus variants in one batch of the same farm confirms the existence of variants independently of one another.

## 4. Discussion

Viral diseases are one of the biggest reasons of the economic problems in aquaculture, causing high losses to fish producers. Although fish pathogens are a well-known problem, reports on the sturgeon virosis are quite limited and mainly concerns American species. Data from Europe have appeared only in recent years. Several viral agents are known to infect sturgeons and the best recognised and most common ones are Sturgeon Nucleocytoplasmic Large DNA Viruses (sNCLDV), representing the most numerous group of viral agents causing disease in many sturgeon species. Most of them have been identified as “iridovirus”, however, only ranavirus Frog Virus 3 (FV3) is an official member of Iridoviridae [6]. The other sNCLDV include the following: White sturgeon iridovirus (WSIV), Missouri River sturgeon iridovirus (MRSIV), Shortnose sturgeon virus (SNSV), British Columbia white sturgeon virus (BCWSV) and Namao virus (NV). These sNCLDV are not yet officially recognised by the International Committee on Taxonomy of Viruses (ICTV), but have shown closer similarity with Mimiviridae, which have shown closer similarity with Mimiviridae as Acipenser iridovirus-European (AcIV-E) [13,18]. Acipenserid herpesvirus 1 (AciHV-1) and Acipenserid herpesvirus 2 (AciHV-2) are species belonging to the Alloherpesviridae family and are the two herpesviruses most frequently reported in sturgeons. AciHV-2, which has been reported in seven species including the Russian and Siberian sturgeon, appears to be particularly dangerous and causes serious health problems [29,30,31]. Other viruses, such as White Sturgeon Adenovirus 1 (WSAdV-1), do not appear to be a problem in sturgeon farming and have only been identified in white sturgeons in America [6]. This work is the first attempt to describe the prevalence of emerging mimivirus and herpesviruses infection in sturgeon farming in Poland.

The presence of mimivirus was confirmed in both healthy and infected fish belonging to two species: the Siberian and Russian sturgeon. The observed clinical symptoms (pale gills and liver, haemorrhages on the skin, abnormal behaviour and increased mortality) were consistent with those in other European countries, such as France, Italy, Ukraine and Sweden, where the mimivirus, also called AcIV-E, occurred [13,14,15,32,33]. Parasites and bacteria were excluded as possible causes of the disease. It is therefore likely that this virus was at least partially, if not fully, responsible for the pathological condition of sturgeons from farm 5 and farm 8. The virus was detected in six farms but three of them had relatively low viral load (Ct > 28, data not shown). However, high Ct values may be due to the fact that the tested fish were clinically healthy and in good condition and were only confirmed as virus carriers. In the other three farms (farm no. 4, 6 and 7), the virus was not detected, but only one sample per farm and a small number of fish were then analysed. Overall, the sample selection in this study was limited due to the small number of farms in Poland and the price of the fish. Therefore, the presence of mimiviruses and the related disease should be more intensively investigated in other farms in Poland, especially since the percentage of infected fish ranged on average from 15 to 33% annually. There have been reports of the presence of WSIV iridovirus in sturgeon in Poland, but the data presented in them were very sparse and unclear [23,24]. In total, they concerned only 29 fish of various species of sturgeon caught in Polish waters or farms and among them there were only one Siberian and three Russian sturgeons. In all cases, the presence of WSIV was confirmed by the PCR method described by Kwak and co-workers [34]. In situ hybridization was also used in this study, but it is not recommended for diagnostic purposes because of its low sensitivity [6,23]. Unfortunately, there is no support for this results in the form of sequences available in GeneBank. In the future, it will be worth it to compare and analyse all the available data, but for now the authors cannot relate to it. In Poland, there is no obligation to test sturgeons for the presence of viral pathogens when transporting from farm to farm or before restocking rivers. It is even more disturbing that one of these reports also mention the presence of WSIV in *A. oxyrinchus,* which was caught from open water and from a farm producing material intended for restocking for the sturgeon restoration program [24]. As sturgeon farms are located along rivers, there is a high risk of the virus spreading with water or with fish being released as part of restocking or trading [35]. Mimivirus can pose a serious threat to the Atlantic sturgeon restitution program in Poland if other species, including the Siberian and Russian sturgeon, will be reared on participating farms.

When it comes to phylogenetic analyses based on the partial MCP sequences, two major mimivirus/AcIV-E lineages were found in Polish samples. Compared with other European sequences, the Polish sequences showed the highest similarity to isolates detected in France and Ukraine [15]. These observations strongly suggest the possible origin of the viruses from both Eastern and Western Europe. There is a possibility that they all can also come from one source, which for these countries could have been fish imported from Russia several decades ago [5,13]. The viruses most likely spread through the trade of contaminated eggs or fish. The presence of two viral lines in a sample at one farm in Poland suggests that they could have been imported many years earlier and mixed, for example, during the process of eggs fertilization. Unfortunately, the analyses of NJ and ML in this particular case show differences in the similarity between the isolate from France and the sample from Ukraine, which may suggest that both variants are mixed and widespread in Europe for a long time [13,15].

The lack of cytopathic effect on the cell line and the negative results of PCR methods for herpesviruses confirm that in the tested samples there were no AciHV-1 and AciHV-2. Additionally, by obtaining a positive signal of the presence of the 16S host gene by Real time PCR, the authors confirmed that DNA extraction provided good quality material and further analysis and a negative result was correct. Herpesvirus infections have been described in the literature in America and Europe [29,30,31]. This rendered the herpesviral infections, such as sNCLDV which are now also called mimiviruses, a major viral diseases in farmed and wild sturgeon. There are no specific molecular tests for the detection of AciHV-1 and AciHV-2, although generic PCR may be useful for their identification [7,8]. Acipenserid herpesvirus 1 (AciHV-1) was the first herpesvirus identified in sturgeons in 1989 in *A. transmontanus* [25]. AciHV-1 produced syncytia and was isolated from a specific monolayer of sturgeon epithelial cells (WSSK-1). The attempts to isolate herpesvirus on this line in our study produced negative results. Other cases of AciHV-1 in white sturgeon have been reported in America and Europe (Italy) [8,29]. These observations are reported in phylogenetic studies and do not describe the symptoms of the disease. Negative results on cell line and PCR test in our study may have resulted in the fact that AciHV-1 has not been confirmed so far in Siberian and Russian sturgeons and these species are not carriers of this pathogen [6]. AciHV-2 was first recorded in 1991 in white sturgeons (*A. transmontanus*) in California [29]. Later, there were reports of the spread of the virus in the United States and Canada and the expansion of the host spectrum (*A. fulvescens* and *A. brevirostrum*) [7,9]. AciHV-2 has been confirmed so far in several species of sturgeon, including *A. beari* in North America and in Europe (Russia, Finland and Kazakhstan) [16,17,30,31]. The authors, therefore, expected that this pathogen may be present in the tested samples from Poland. Fortunately, our suspicions were not confirmed. This pathogen was not found in samples from Polish farms nor even in those Siberian sturgeons from farm 5 that showed clinical symptoms similar to those mentioned in the literature. The AciHV-2 has not yet been observed in the Russian sturgeon species [6]. It appears to be restricted to the host sturgeon species and mainly occurs in Eastern North America or Russia as closely related to the specific species of fish [6]. Due to CITES convention for species protection, sturgeons from Poland have no natural contact with North American or Russian species. To the best of our knowledge, there has been no official direct trade in wild live fish between the countries of North America or Russia and Poland for at least twenty years. The only method that could allow the pathogens to be transmitted was importing of eggs derived from the population of Atlantic sturgeon (*A. oxyrinchus*) inhabiting the St. John River on the coast of Canada for the purpose of the sturgeon restitution program in Poland [5]. Currently, AciHV-2 has been found in this river in Canada and only in the species *A. brevirostrum* [29]. It should be mentioned, however, that these findings do not exclude the possibility of herpesvirus introduction to Poland with the transport of fish or eggs, but their presence has not yet been discovered.

## 5. Conclusions

In conclusion, our study provides the first evidence of the emergence of mimivirus in Siberian and Russian sturgeon farms in Poland. The average level of infection on farms per year was within the range of 15–33% and the more samples were tested, the lower it was. A mixture of the two variants of mimivirus was confirmed, although the European variant was clearly dominant in Poland. However, herpesviruses were not detected in the tested samples, which is an optimistic conclusion from this study and, at the same time, motivates further research in this area. Considering the growing importance of the sturgeon meat and caviar production in Poland, which is the third producer of caviar in the world, it is recommended that general health surveillance tests are implemented, especially for viral diseases and the improvement of biosecurity measures in order to avoid the spread of pathogens threatening the sturgeon restitution program in Polish waters.

## Figures and Tables

**Table 1:** Sequences of the oligonucleotides and probes used in this study.

Method	Sequence	Source
	**Host 16S rRNA**	
**Real Time** **PCR**	Aci16SFw: AAGGAACTCGGCAAACACGA Aci16SRv: GCTACCTTCGCACGGTCAAA Aci16SPb: Cy5-AGAGGTCCCGCCTGCCCTG-BHQ2	[15]
	**Mimivirus**	
**cPCR set A** **cPCR set D**	F-oPVP339: GATAGGGTACAAGAGACATTC R-oPVP340: TCAACCAAATATTCTTGCTTG F-oPVP339: GATAGGGTACAAGAGACATTC R-oPVP342: TCAACCAAATATTCTTGCTTG	[13]
**Real Time** **PCR**	F-oPVP346: TCAAAGTCTGGGACCTCTA R-oPVP347: AGAGATGTTCAACTGGATGT tqPVP20:FAM-TTGTGAATCATATCGCCAGTCAT-BHQ1	[13]
	**Herpesvirus**	
**cPCR AciHV-1** **cPCR AciHV-2**	F-308: 5′-ACCTCGTGTTGATCG-3′ R-309: 5′-TCAAAACTTCCGGGT-3′ TermF2 5′-GCMMGRGGACAGAWCCCMG-3′ Termsal-3Rdeg 5′-GGTGCACACRCCMADIGACG-3′,	[8]

**Table 2:** Fish samples description. (S) Samples sequenced for the genetic analysis of mimivirus.

Number of Farm /Year of Sampling	Description of Fish	Number of Sample	Number of Samples Positive/All	Number of Positive Samples (Real Time)	GenBank Accession Number
1/2016	Siberian sturgeon 1000–2000 g, 47 fish	PL1601-PL1647	7/47	1601 (s), 1602 (s), 1603 (s), 1615, 1616, 1625, 1626	MG212658, MG212659, MG212660
2/2016	Siberian sturgeon 5–10 g, 6 fish	PL1648-Pl1653	1/6	1650	-
3/2017	Siberian sturgeon 500–700 g, 12 fish	PL1701-PL1712	6/12	1706 (s), 1702, 1703, 1704, 1705, 1708	MG212661
4/2017	Siberian sturgeon 15–50 g, 6 fish	PL1713-PL1718	0/6	-	-
5/2017	Siberian sturgeon 30–50 g, 11 fish	PL1719-PL1729	8/11	1719 (s), 1719.1 (s), 1720 (s), 1720.1 (s), 1721 (s), 1721.1 (s), 1722, 1726	MG212662, MG212663, MG212664, MG212665, MG212666, MG212667
6/2017	Siberian sturgeon 20–50 g, 10 fish	PL1730-PL1739	0/10	-	-
7/2017	Russian sturgeon 10–15 g, 10 fish	PL1740-PL1750	0/10	-	-
8/2018	Russian sturgeon 1000–1500 g, 6 fish	PL1801-PL1806	2/6	1801 (s), 1802 (s)	MN542940, MN542941
9/2020	Siberian sturgeon 10–50 g, 28 fish	PL2001-PL2028	6/28	2001 (s), 2002 (s), 2003 (s), 2004 (s), 2005 (s), 2006 (s)	MT735127, MT735128, MT735129, MT735130, MT735131, MT735132

## Data Availability

Not applicable.
